# The Hospital World

**Published:** 1911-09-09

**Authors:** 


### THE HOSPITAL WORLD.

### A Statuette for Worcester Infirmary.

AT the August monthly meeting of the Worcester General Infirmary's Executive Committee, the Chairman announced that Mrs. Littleton Wheeler had presented a statuette of the "Good Shepherd" to the "Bonaker" Ward, as a memento of her husband, the late Rev. Canon Littleton Wheeler. A vote of sincere thanks to the donor was passed, and the ward is much flattered to have been chosen for a present which will distinguish it so gracefully from its fellows.

### Sudden Death of a Young Surgeon.

THE sudden death is reported of Mr. David Kirkpatrick Coutts, F.R.C.S., of Norwich, in his 30th year. Mr. Coutts only qualified seven years ago, after a brilliant career as a student of St. Thomas's Hospital. He held several appointments in his hospital, including that of clinical assistant in the throat department, and house appointments. He was also assistant lecturer on Practical Surgery and Prosector to the Society of Apothecaries. He took successively the degree of M.B., B.S., of the London University in 1905, and the Fellowship of the Royal College of Surgeons in 1906. Mr. Coutts afterwards went to Cairo, where he held the post of resident surgical officer at the Kasr-el-Ainy Hospital. In Norfolk he had already a promising surgical practice, and his appointments included those of honorary consulting surgeon to the Cromer Cottage Hospital, and to the Victoria Hospital, Swaffham. Only a few months ago he was elected Assistant Surgeon to the Norfolk and Norwich Hospital, where he found scope for his great surgical aptitude. His untimely death at his home in Norwich on August 21 took place with painful suddenness and while he was apparently in good health. Sincere sympathy will be felt for his wife in her tragic bereavement.

### The late Mr. Rainy, M.B., M.P.

THE early death of Mr Adam Rolland Rainy, which took place suddenly on August 26, at his residence in North Berwick, removes one of the small band of medical men in the House of Commons. Mr. Rainy was born in 1862, and took the degrees of M.B., C.M., at Edinburgh University, in 1886. He practised in London as an ophthalmologist until 1900, in which year he gave up medical for political work. He contested Kilmarnock Burghs as a Liberal in the latter year, but unsuccessfully. In 1906, however, he won the seat, and held it by increasing majorities at subsequent elections. Mr. Rainy was a son of the well-known Presbyterian leader, Principal Rainy, and was himself a trusted exponent of that denomination. It seems too much to hope that his place in the House may be filled by another medical man; but in view of the detailed discussion of the Insurance Bill, which has still to be undertaken, it is most unfortunate that there should be any diminution in the number of professional critics and advisers in debate. Dr. A. R. Rainy was one of the Presidents of the Diagnostic Debating Club, an office which his father had filled nearly forty years before him.

### Another Early Death in the Profession.

THE death after an illness of three days' duration is reported of Dr. John Henry Harbinson. He qualified at the Queen's University, Belfast, in 1910 as M.B., B.Ch., having been a distinguished intermediate exhibitioner. He held the appointment of house surgeon at the Mater Misericordiæ Hospital, Dublin, and proceeded to the post of demonstrator in physiology at Queen's College, Belfast. His sudden death occurred in Liverpool, and is a source of grief to his numerous friends among the junior members of the profession in Belfast.

### A Rector and Chesterfield Hospital.

THE trustees of the late Mr. H. J. Morgan, some time Rector of Eckington, a parish in the area served by the Chesterfield and North Derbyshire Hospital, have sent to the Treasurer, Mr. C. J. Howson, the sum of £500 as a legacy to the institution. This legacy, which is free of duty, will, it is hoped, stimulate the efforts of the local subscribers, being, as it is, the practical example set by a parish priest who must often have upheld the claims of Hospital Sunday. Mr. Morgan's testamentary generosity should stimulate the local clergy of all denominations when the next Hospital Sunday comes round.

### A Loss to Peterborough Infirmary.

DR. LEONARD CANE, a well-known practitioner of Peterborough, has died at Eastbourne in his sixty-fourth year. He had not long relinquished practice in Peterborough, being succeeded in the firm of Cane and Latham by Dr. Leonard Buckell Cane. He was a student of University College, London, and qualified in 1871; the following year he obtained honours in the M.B.(Lond.) and also took the B.S. degree and the conjoint diploma. Dr. Cane became M.D.(Lond.) in 1873. He was a keen and hard worker, and published several papers on surgical subjects in the *Lancet*. In 1871 he carried off the Liston prize for an essay on "Antiseptic Surgery" at a time when this new method was the subject of much controversy. For many years he was on the staff of the Peterborough Infirmary, to which he was a consulting physician at the time of his death.

### A Nicaraguan Practitioner.

THE death has occurred in Nicaragua of Dr. Francis Charles Browne-Webber, at the age of fifty-six. He was the son of the late Valentine Browne-Webber, M.D., of Darjeeling, and studied at the Catholic University of Dublin, taking the degree of M.B. (R.U.I.) in 1886. Dr. Browne-Webber had been for many years settled in Nicaragua, and had obtained in 1900 the M.D. of the University of Leon. He was pathologist to the hospital at Managua, Inspector-General of the pharmacies of the Republic, and Government Chemist of Nicaragua. At the University of Azuay he was formerly professor of bacteriology and histology, and had published studies on the marsh fevers of the country.

### A Former Indian Medical Surgeon.

SURGEON-MAJOR JOHN INCE, M.D., late of the Indian Medical Service, has died at his residence near Swanley, Kent, at the age of eighty-one. Major Ince was an old Guy's man, qualifying as M.R.C.S.Eng. in 1854, and took the M.D. of St. Andrews in the following year. Before proceeding to India he held the post of resident surgeon at the Surrey Dispensary for nearly two years. In 1864 he was medical officer in Kashmir, and was later civil surgeon in Murree and Rawalpindi. Major Ince was a Fellow of a large number of learned societies, and was a member of the Council of the Hospitals Association. His literary work was extensive, and included several official reports on epidemic cholera in Peshawar in 1869, and more recently on the M.A.B. smallpox camps in 1885. He was the author of the "Murree Directory" and the "Murree Handbook," and his "Kashmir Handbook" attained a fourth edition in 1892. Between 1872 and 1874 Major Ince undertook investigations on the prevalence of typhus and typhoid fevers in the gaols of India.

